# Effectiveness of the WHO SCC on improving adherence to essential practices during childbirth, in resource constrained settings

**DOI:** 10.1186/s12884-016-1139-x

**Published:** 2016-11-08

**Authors:** Somesh Kumar, Vikas Yadav, Sudharsanam Balasubramaniam, Yashpal Jain, Chandra Shekhar Joshi, Kailash Saran, Bulbul Sood

**Affiliations:** Johns Hopkins University, 221, Okhla phase 3, New Delhi, 110020 India

**Keywords:** Safe Childbirth Checklist, Intervention, Practices, Low resource settings

## Abstract

**Background:**

India accounts for 27 % of world’s neonatal deaths. Although more Indian women deliver in facilities currently than a decade ago, early neonatal mortality has not declined, likely because of insufficient quality of care. The WHO Safe Childbirth Checklist (SCC) was developed to support health workers to perform essential practices known to reduce preventable maternal and new-born deaths around the time of childbirth. Despite promising early research many outstanding questions remain about effectiveness of the SCC in low-resource settings.

**Methods:**

In collaboration with the Ministry of Health SCC was modified for Indian context and introduced in 101 intervention facilities in Rajasthan, India and 99 facilities served as comparison to study if it reduces mortality. This Quasi experimental Observational intervention-comparison was embedded in this larger program to test whether a program for introduction of SCC with simple implementation package was associated with increased adherence to 28 evidence-based practices. This study was conducted in 8 intervention and 8 comparison sites. Program interventions to promote appropriate use of the SCC included orienting providers to the checklist, modest modifications of the SCC to promote provider uptake and accountability, ensuring availability of essential supplies, and providing supportive supervision for helping providers in using the SCC.

**Results:**

The SCC was used by providers in 86 % of 240 deliveries observed in the eight intervention facilities. Providers in the intervention group significantly adhered to practices included in the SCC than providers in the comparison group controlling for baseline scores and confounders. Women delivering in the intervention facilities received on an average 11.5 more of the 28 practices included compared with women in the comparison facilities. For selected practices provider performance in the intervention group increased as much as 93 % than comparison sites.

**Conclusion:**

Use of the SCC and provider performance of best practices increased in intervention facilities reflecting improvement in quality of facility childbirth care for women and new-born in low resource settings.

## Background

Achieving the desired reduction in preventable maternal and child mortality remains the unfinished agenda of the Millennium Development Goals (MDGs) [1–4]. This has remained unachieved despite knowing what works for reducing maternal and child mortality in developing country contexts for many years [5, 6] It has been estimated that globally, better care during labor and birth, and care of new-borns immediately after birth can avert up to 1.49 million maternal and new-born deaths and still births.(6) There is an urgent need to fill the gap between evidence and its translation to practices during care provision, particularly in developing country settings.

Nowhere is the need for filling this gap greater than in India. With the burden of 0.76 million neonatal deaths, India tops the list of countries with high Neonatal Mortality Rate (NMR) [7]. India has infant and under-five child mortality rates of 42 and 52 per 1000 live births respectively, and about 70 % of infant deaths and more than half of under-five child deaths in India occur in the neonatal period, the first 4 weeks of life [8].

While the infant mortality rate (IMR) has declined steadily, early neonatal mortality rate (ENMR) has virtually remained static since the last decade [9]. Considering the fact that neonatal deaths account for up to 40 % of under-5 deaths [3], the need for focusing on perinatal care in India is an urgent local and global priority.

The three-delay framework (decision to seek, reaching, receiving adequate care), used for understanding maternal and newborn mortality in developing countries applies well to the Indian context for maternal and newborn mortality [10–12] Acting on the best available evidence that having mothers deliver in institutions rather than homes improves birth outcomes, India invested heavily on improving institutional delivery rates through programs such as Janani Suraksha Yojna (JSY a conditional cash transfer program for women delivering at institutions.). Rates of institutional delivery have more than doubled since the advent of National Rural Health Mission (NRHM), and currently more than 74 % of total births in India are occurring at institutions [13]. However, various studies have failed to show a commensurate reduction in maternal and neonatal mortality [14, 15]. Thus, just having a contact with the health system for deliveries has not proven to be enough for improving outcomes, which suggests a need to focus on the quality of care during these contacts. Experience from JSY also indicates that while the focus on bringing women to institutions may have influenced the first and second delay in care, the third delay, i.e., in timely and high-quality care provision at institutions is still a challenge [14].

It is well known that the capacity for quality of care for maternity services is influenced by skills of health workers in performing essential practices, availability of essential resources, presence of appropriate and evidence-based guidance for action, and an overall enabling environment including effective organization of health care services [1–4]. In the Indian context, as in many settings, accountability and motivation of health workers to translate this capacity for quality into action are additional important influencers of quality of care. The Indian government has developed many guidelines focusing on quality of perinatal care and has invested resources on training health workers through skill building programs for childbirth related care [16, 17]. However, these initiatives have not translated into improved quality of care. A recent study in Delhi points out the widespread non-adherence to evidence-based practices in both public and private sector maternity care institutions [18]. Thus, in the Indian context, there is a strong felt need to develop and test tools and technologies that can help health workers to translate evidence into action.

The WHO Safe Childbirth Checklist has been considered as a promising frugal technology aiming at improving childbirth related care [19]. Building upon the success of the Surgical Safety Checklist in improving quality outcomes in health practices [20], this checklist was developed by WHO to support health workers to perform essential practices and prevent avoidable childbirth-related deaths [21]. The SCC targets high impact best practices around 4 pause points that occur in almost every delivery: admission, pushing, just after delivery and pre-discharge. In a single facility study in Karnataka, the use of SCC significantly improved the delivery of essential safety practices by health workers during childbirth [22]. However, while the study provided initial evidence on the effectiveness of SCC for improving service quality, it reported limitations regarding generalizability of and sustainability of findings due to the study design, nature of intervention, and setting of the study [22]. Considering the urgent need for India to identify solutions to improve quality of childbirth care at scale, evaluation of interventions that promote uptake and adherence with a simple technology like the SCC within realistic program implementation settings represents an important research priority for the country. Moreover, the WHO SCC multi-country collaboration is keenly interested in implementation experience and evidence from multiple settings to generate guidance on how the SCC should be used on a global scale [23].

Responding to the needs described above, a large quasi-experimental design study was conducted in 200 health facilities in the state of Rajasthan in India to understand the effectiveness of the SCC on childbirth-related outcomes within the ‘real-world’ program and administrative framework in the country. While the larger study was designed with a view to capture effects of SCC use on client outcomes, a smaller sub-study was conducted to understand the pathway by which the SCC changes these outcomes. Through direct observations in the labour rooms, we tried to assess effectiveness of SCC in bringing changes in adherence to safe care practices by the health workers in intervention facilities as compared to the comparison facilities. In this paper, we are reporting the results of this observational study, nested within the larger quasi-experimental design study, to assess whether women receive more life-saving practices during labour, delivery, and the immediate postpartum period in facilities where SCC was introduced compared to women in facilities where the SCC was not introduced. A separate evaluation is going to assess the impact of the SCC use on in-facility perinatal mortality rates.

## Methods

### Study design and setting

This is a quasi-experimental observational study implemented in the state of Rajasthan in India from March 2013 to April 2014.

This study compared health providers’ use of the SCC and adherence with SCC items in 8 intervention and 8 comparison facilities at the baseline (March- April 2013) and end line (March- April 2014). Intervention and comparison facilities included two district hospitals and six Community Health Centres (CHC)/Sub-District Hospitals (SDH) in each arm, from the intervention and comparison districts matched by average annual delivery load. These facilities were selected randomly from the overall available sample of 13 District Hospitals and 187 CHC/ SDHs.

### Intervention

The Government of Rajasthan implemented the SCC program at Community Health Centers (CHC), Sub-district Hospitals and District Hospitals of selected districts with technical support from Jhpiego. Technical assistance of Jhpiego was aimed at pilot testing of SCC, modification in SCC to adapt to the local context, orientation of providers, facility readiness assessments, and conducting post-training follow-up and support visits at pre-defined frequency.

Several modifications to the SCC were informed by an initial pilot test of the SCC and input of a Technical Advisory Group (TAG). Modifications included introduction of a designated space to record maternal and newborn vital signs (e.g., mother’s temperature and blood pressure, newborn’s weight). The modified SCC included 31 critical practices and retained the original four pause points distributed across two pages. For this checklist there were four pause points: at admission, just prior to or during pushing, immediately after delivery and prior to discharge. But of this 31 practices only 28 were observed during this study and mentioned in the results.

Prior to the implementation program staff assessed availability of essential drugs, supplies and functionality of equipment required to implement SCC practices in both intervention and comparison facilities. Essential commodities and equipment identified as missing during assessments were provided by the program for both intervention and comparison facilities. Availability of supplies was monitored periodically in both intervention and comparison facilities.

The SCC program included two main components-a 1.5 day orientation of facility-based health workers on using the SCC as a part of their care delivery and post-training on-site support in using the SCC.

After the training, the health workers in intervention facilities attached the SCC to the existing case-sheets of the individual patient. It is important to note that at the time of intervention, templates of case-sheets were already in use at both intervention and comparison facilities. For implementation purposes, the SCC was externally attached to the existing case-sheets in intervention facilities. Case-sheets in comparison facilities remained un-changed.

After the initial training, Jhpiego staff made site visits to the intervention sites on a set frequency. to assess the progress of SCC use in the facility and to address barriers to its smooth implementation of the checklist. The first on-site visit was made to a facility where orientation of all health workers has been completed within 15 days of the completion of orientation. Fortnightly visits continued up to 2 months. After 2 months, the frequency of visits was reduced to once a month for next 6 months. After 6 months, the facilities were visited at least once every quarter for the remaining duration of the study. Visits typically included the following activities: observation of SCC use by on-duty health workers and onsite support in effectively using it, facilitation of the availability of the SCC at the sites, and any administrative barriers to the health workers in using the SCC. Apart from this, Jhpiego staff also facilitated all-staff meetings in the intervention facilities at least once every quarter as a part of the onsite visits where all relevant health workers from the facilities came together and discussed their experiences with SCC use; challenges faced, and suggested remedies to these challenges.

An important characteristic of the SCC program was that implementation approach was developed in close collaboration with the Ministry of Health and was firmly grounded in the existing public health system. For example, resources of the public health system were used to support most implementation activities such as provider orientations. Existing Skilled birth attendants (SBA) trainers in the public health system were used to orient the providers on the SCC, at training sites which were mostly public sector district hospitals of the state. As far as possible, the system’s resources were leveraged to ensure availability of essential supplies in intervention and comparison facilities.

### Sample size

The sample size calculation was based on the conservative assumption that the essential practices would be done in 50 % of deliveries, and having 80 % power to detect a 20 % increase in practice coverage. Alpha was set at 0.0017 using Bonferroni’s correction considering 28 comparisons. The design effect-adjusted group sample size in comparison and intervention arm was found to be 20 observations per group considering 0.01 intra-class correlation coefficient. This resulted in 960 observations overall in each comparison and intervention arm, total sample size was 240 per pause point per arm. Some clients were observed in more than one pause point.

### Sampling method

Every woman delivering in a facility during observation period who was not in active labour and was an appropriate state of mind to consent were approached and those who consented were included in the study until the required number of observations were achieved for a pause point in a facility.

### Data collection tools

The number of providers available and the delivery load initial infrastructure, supplies and training status of staff available were collected during baseline using a rapid facility assessment tool. A Periodic assessment tool which had information about the facility type, average delivery load, staff available and supplies was also used every quarter to assess the resource availability and practices. Training data was available from training data formats which updated data as trainings happened. The details of supplies came from the monthly reports of facilities which reported on availability of number of supplies.

Observers used pre-designed structured observation format to collect data on different pause points, which was supplemented by a self-contained step by step procedure guide (Algorithm). This Algorithm clearly defined the practice to be performed by a provider was performed or not. For example for Appropriate hand hygiene is considered to be performed only if Provider had access to running water, soap, gloves and performed six steps of hand washing before each internal examination and wore gloves.

### Procedures

Each facility was approached for permission to observe the deliveries. Observers were typically a Graduate Nursing (BSc) school student/intern who had basic knowledge of maternal and childbirth-related practices. Observers were oriented for one day on the checklist and how to use a standardized algorithm to classify practices as being performed, not being performed or not applicable. Supervised observations in the labor room (at least once for each of observers) and 2 days mock sessions were also part of the training. The observers worked in round-the-clock shifts of 8 h each till the time the required sample size was achieved for one facility. Observations were made in the facilities only after at least 6 months were completed after the initial introduction.

The unit of observation was a pause point rather than a delivery. One delivery (if followed from the period of admission to discharge) made for four independent pause point observations. 240 observations were made for each pause point. Each data collector continuously observed care for a pregnant woman at all the pause points applicable to his/her shift at the facilities. These observations lasted for the whole duration for which the provider completed activities relevant to that pause point. Apart from observing practices, the data collectors also observed the SCC use for that pause point. In addition, they made periodic observations and record checks to confirm where activities such as use of partograph were completed. At each pause point, they observed relevant practices where they were performed (admission room, labor room, and post-partum ward). For efficiency of operations, they prioritized observing different pause points on all available cases during one shift rather than following one case from point of admission to discharge.

Providers were recruited in their free time in a private space. Observers explained the study purpose and process to the providers that they would be observed for their practices at four different pause points and obtained informed oral consent at the beginning of the baseline and endline data collection.

Informed oral consent of mothers was taken at the admission for observing her at various points of her child birth using a local vernacular consent form. If the observer was a male, presence of a female attendant was ensured during observation. Mothers who did not consent, were in severe pain, or were in a state in which they were unable to consent were not included for observation.

Each practice was observed at every pause point and was categorised based on the algorithm as practiced, not practiced and or not applicable. Data quality was assured by using standardised formats and algorithms, standardised training, periodic review and mentoring of observers and data quality checks in data entry. The completed observation forms were entered in CS-Pro and the data was cleaned for inconsistencies using data validation and internal consistency.

### Data analysis

Data was analysed using MS excel 2010, Stata version 13.0 and SPSS version 22.0. Frequencies and categorical analysis was done for the cleaned data. Proportions were calculated for all categorical variables. Chi-square test was performed for statistical significance of proportions. A composite index was developed for availability of supplies and equipment based on the availability of such supplies during the time of the study. which was used for the regression analysis. Multiple Linear Regression analysis was done to find the difference in difference (DID) in average number of practices performed during intrapartum and postpartum care in intervention and comparison facilities between baseline and endline. The model estimate was created with robust standard error and considering the clustering at client level and the facility level and adjusting for health worker type and availability of supplies and drugs. Difference in difference of means was determined by an interaction term in the regression model between intervention/comparison and baseline/endline (time) variables. A difference in difference logistic regression analysis was performed for individual practices and DID estimator-which was the interaction term in the model-was calculated for each of the practices. This DID estimator was adjusted for clustering by health worker, and a composite index for supplies, health worker categories.

*P* value less than 0.0017 was considered statistically significant considering Bonferroni’s correction.

### Ethical considerations

This observational study was reviewed and approved by Government of Rajasthan and the institutional review board (IRB) of Johns Hopkins Bloomberg School of Public health, Baltimore, USA (IRB 0004816). Verbal Consent of providers and mothers was obtained for participation in this study as per the protocol approved by IRB. Written consent was not obtained as we did not want to record any identifiable information of the client or the provider. Consent was obtained by allowing the study participant to read the consent form in local vernacular language or loudly read the consent forms to the study participant and the person who administers the consent signed it and left a copy of the consent with the study participant.

## Results

The SCC was used in 86 % of the observed deliveries in intervention facilities, which implies successful adoption by a majority of providers in the intervention facilities. Table 1 presents basic background information of the intervention and comparison facilities including workload, human resources and supplies. The facilities in each arm were similar with respect to delivery volume and the number of staff at baseline and endline. The composite index of supplies and equipments increased in intervention and comparison sites to 0.9 at end line. 63 % (12/19) of the total doctors trained on SCC at baseline and 92 % (58/63) 92 % of the nurses trained on SCC at baseline were available in intervention facilities at the end line.

**Table 1:** Background information of the facilities in which the safechild birth study was conducted during baseline and endline

Observational Study	Type of facility			Average monthly delivery load		Staff Available		Staff Oriented on SCC		Supply Composite Index
	DH	SDH	CHC	DH	CHC/SDH	Doctor	Nurse	Doctor	Nurse	
Baseline Intervention	2	1	5	462	159	21	56	0	0	0.69
Baseline Control	2	1	5	339	134	23	52	0	0	0.75
Endline Intervention	2	1	5	388	138	19	60	11	55	0.9
Endline Control	2	1	5	277	129	27	55	0	0	0.9

Table 2 illustrates the distribution of clients assessed/attended at the study sites by cadre. In intervention sites 15 % of the women who delivered at the facilities were attended by doctors, 80 % by nursing staff and 5 % by others. In comparison sites the provider cadre distribution was similar. In both groups a larger proportion of providers were doctors at discharge than at any other pause point whereas in others it was predominantly nurses.

**Table 2:** Percentage of pregnant women assessed by providers at each pause point in end line in observational study

Pause points	Intervention			Comparison		
	Doctors	Staff nurse	Others	Doctors	Staff nurse	Others
On Admission (*N* = 240 Deliveries)	14 %	82 %	5 %	18 %	75 %	7 %
Just before pushing (*N* = 240 Deliveries)	10 %	84 %	6 %	6 %	84 %	10 %
Soon after birth (*N* = 240 Deliveries)	9 %	85 %	6 %	8 %	84 %	8 %
On Discharge (*N* = 240 Deliveries)	28 %	70 %	3 %	32 %	65 %	3 %
Overall (﻿*N*=960 ﻿Deliveries)	15 %	80 %	5 %	16 %	77 %	7 %

Table 3 provides a comparison of practices across all pause points in intervention and comparison facilities during the baseline and endline. In intervention sites, the difference in the number of practices observed at endline compared to baseline was statistically significant for 26 of the 28 practices and 21 of the 28 practices were observed over half the time. Only assessment of breathing at one minute and recording of baby’s birth weight did not change significantly, and both of these practices were already observed at least 95 % of the time at baseline. In the comparison group 9 of the 28 practices improved significantly from baseline to endline, however, only four practices were being done more than half of the time.

**Table 3:** Univariate and Multivariate analysis of Provider’s adherence to safe child birth checklistpractices (based on 240 observations per pause point during baseline and endline in intervention and control facilities)

Practices	Intervention		*P* value	Comparison		*P* value	*P* value
	Base line	End line	Baseline Vs Endline in Intervention	Base line	End line	Baseline Vs Endline in Comparison	DID modeling-Logistic regression
On Admission							
Assessment and appropriate referral	2 %	88 %	<0.001	3 %	7 %	0.036	< 0.001
Partograph used	13 %	52 %	< 0.001	8 %	0 %	< 0.001	< 0.001
Appropriate Maternal Infection management	0 %	76 %	< 0.001	2 %	8 %	0.008	< 0.001
preeclampsia management	35 %	74 %	< 0.001	14 %	15 %	0.365	.001
HIV tested	16 %	56 %	< 0.001	32 %	29 %	0.314	< 0.001
Companion briefed on danger sign	2 %	66 %	< 0.001	4 %	8 %	0.042	< 0.001
Appropriate hand hygiene	2 %	21 %	< 0.001	0 %	3 %	0.038	0.994
Just Before Pushing (or Before Cesarean)							
Appropriate hand hygiene	2 %	18 %	< 0.001	0 %	5 %	< 0.001	0.972
Oxytocin in one min of delivery	24 %	88 %	< 0.001	32 %	49 %	< 0.001	< 0.001
Cord cut with sterile blade /scissor	8 %	47 %	< 0.001	17 %	32 %	< 0.001	0.008
Assessment of baby breathing in one minute.	95 %	97 %	0.21	94 %	95 %	0.179	0.702
Appropriate NB thermal management	4 %	98 %	< 0.001	16 %	75 %	< 0.001	< 0.001
Appropriate NB Resuscitation^a^	41 %	83 %	< 0.001	29 %	30 %	0.623	0.026
briefing for birth helper in EM	10 %	78 %	< 0.001	15 %	32 %	< 0.001	0.008
Soon After Birth(within 1 h)							
Blood loss assessed in mother	35 %	91 %	< 0.001	49 %	68 %	< 0.001	< 0.001
Appropriate maternal infection management	1 %	74 %	< 0.001	3 %	1 %	0.313	< 0.001
NB Assessed for antibiotics	1 %	43 %	< 0.001	4 %	4 %	0.027	< 0.001
Birth Weight taken	99 %	98 %	0.284	85 %	78 %	0.026	0.665
Initiated breastfeeding in One hour of birth	34 %	86 %	< 0.001	43 %	46 %	0.224	< 0.001
Skin to skin contact with mother	13 %	37 %	< 0.001	2 %	22 %	< 0.001	0.104
Companion briefed on danger sign (M&NB)	4 %	52 %	< 0.001	3 %	5 %	0.176	< 0.001
Before Discharge							
Appropriate maternal blood loss assessment	9 %	70 %	< 0.001	3 %	40 %	< 0.001	0.694
Appropriate maternal infection management	0 %	72 %	< 0.001	2 %	3 %	0.539	< 0.001
Appropriate NB Infection management	0 %	58 %	< 0.001	0 %	2 %	0.043	< 0.001
NB feeding assessment	13 %	81 %	< 0.001	11 %	12 %	0.834	.001
Follow up advise to mother	0 %	54 %	< 0.001	4 %	10 %	0.009	.002
FP options discussed	5 %	43 %	< 0.001	14 %	8 %	0.067	.003
Discharge counselling on danger sign	0 %	47 %	< 0.001	0 %	6 %	< 0.001	.167

^a^This practice is on observation of less number (baseline intervention = 46, endline control = 101, baseline control = 38, endline control = 46)

When a logistic regression model with robust standard error was run for each of the 28 practices adjusting for covariates of clustering by health worker, and a composite index for supplies, health worker, health worker categories) and testing the significance of the interaction term estimating the difference in difference (i.e., time-baseline and endline-and group-intervention and comparison) 16 of the 28 practices had improved significantly more in the intervention than in the comparison group. During the first pause point partograph use, preeclampsia management, HIV testing of mothers and companion briefed on danger signs were statistically significant (*p* < .001). In the second pause point, giving oxytocin to mothers within one minute of delivery and appropriate new born thermal management were statistically significant. In the third pause point blood loss assessment, appropriate maternal infection management, new born assessment for antibiotics, initiation of breast feeding and briefing companion on danger signs were statistically significant. In the fourth pause point appropriate new born infection management, new born feeding assessment and appropriate maternal infection management were statistically significant. These practices in endline intervention group was higher than all other groups. Since the *P* value of Less than 0.0017 was the cut off due to Bonferroni’s correction the difference in difference of proportion of these practices were statistically significant.

**Fig. 1:**
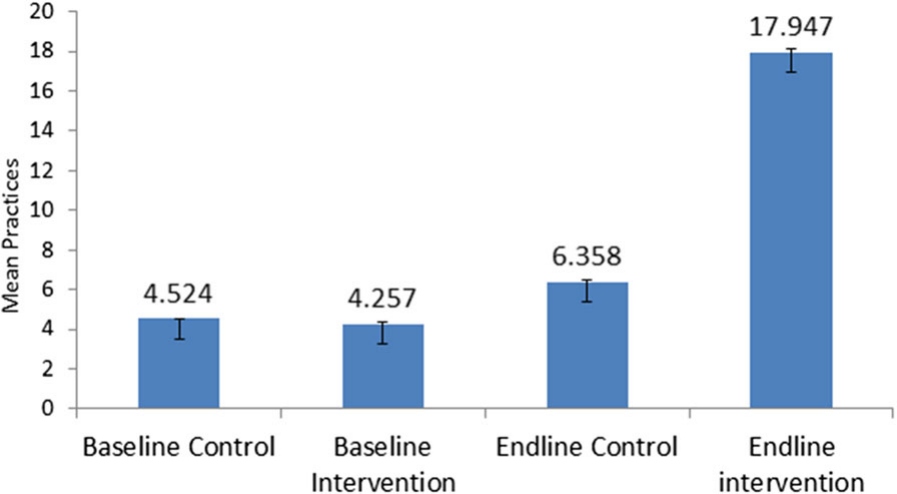
Average number of Practices done by providers at all pause point in observational study

Figure 1 illustrates the average number of evidence-based practices included in the SCC performed on each client in intervention and comparison groups at baseline and endline. The mean number of practices increased from 4.5 practices to 6.4 in the comparison group and from 4.3 practices to 17.9 practices in the intervention group.

Table 4 depicts the multiple linear regressions with robust standard error estimation adjusted for health worker type, facility type, supply index and clustering of observations by health workers. The mean difference in difference in practices at pause point one was 4.0 (95 % CI 3.3–4.8), pause point two was 1.6 (95 % CI 1.1–2.3), pause point three was 2.5 (95 % CI 2.0–3.1) and pause point four was 3.5 (95 % CI 2.3–4.7). All these differences were statistically significant (*p* < .001).

**Table 4:** Linear regression model estimates of difference in difference in mean number of practices at various pause points observed during the Safe child birth checklist study

Pause point	Number of practices	Mean difference in difference of practices (95 % CI)^a^	*P* Value (*t*-test)	*R2* value
At admission	07	4.0 (3.3–4.8)	<0.0001	0.44
Before delivery	07	1.6 (1.1–2.3)	<0.0001	0.62
One hour after delivery	07	2.5 (2.0–3.1)	<0.0001	0.55
Before discharge	07	3.5 (2.3–4.7)	<0.0001	0.65
All practices	28	11.5 (8.5–14.6)	<0.0001	0.76

^a^for a DID estimator by logistic regression with robust standard error and adjusted for health worker clustering for observations, health worker type, supplies and drugs and facility type

On average a client in the intervention facility received 11.5 more SCC (95 % CI-8.5–14.6)) best practices than a client in comparison sites controlling for baseline values; this difference was statistically significant (*p* < 0.001).

## Discussion

In this study, we evaluated the effectiveness of a SCC in ensuring adherence to safe childbirth related practices known to have high impact on reducing preventable maternal and neonatal mortality around the time of delivery as well as an optimal implementation approach for increasing providers’ use of the SCC in ‘real-life’ facility settings. We observed that an overwhelming majority of providers in the intervention facilities did use the SCC during the delivery process. Introduction of the SCC supported by a simple implementation package resulted in a clear improvement in adherence with SCC essential practices in the facilities where it was introduced as compared to the facilities where it was not. Out of the 28 essential practices, women in the intervention facilities received nearly 12 more practices than in the comparison facilities. Given the findings of the study, it appears that introduction of the SCC in association with a light implementation package may be an effective approach for helping to close the “know-do” gap in intrapartum care best practices.

During the study, it was observed that the SCC helped to strengthen the quality of initial assessment and appropriate referral of the women at the time of admission. Inappropriate and delayed initial assessment and referral at the time of admission, classically categorized as the third delay, are major contributors to maternal and new-born morbidity and mortality [10–12]. It is apparent from our observations that the SCC has a good potential to reduce this delay through better assessments at the time of admission.

Adherence to several important maternal clinical care practices proven to reduce the incidence and mortality due to complications like post-partum haemorrhage improved more in the intervention facilities (e.g., adherence with immediate postpartum administration of oxytocin for reduction of PPH.)

Study results also demonstrated increased adherence with several lifesaving interventions for the newborn such as appropriate thermal management which included drying of the baby with dry towel, timely and appropriate resuscitation and immediate initiation of breastfeeding. It is noteworthy that in spite of large amount of resources spent on national training initiatives like trainings for Skilled Birth Attendance (SBA) or the national newborn survival training program (‘Navjat shishu Suraksha Karyakram’-NSSK), the practices related to essential newborn care like immediate initiation of breastfeeding are still sub-optimal in many parts of India [24–28]. Thus the improved adherence with lifesaving practices by the health workers in SCC intervention sites highlights the potential of SCC in helping translate knowledge and skills into practice.

Assessment of the well-being of the mother and newborn both at the time of admission and at the time of discharge also improved significantly more in the facilities where the SCC was used. This reflects the important role of the SCC in standardizing facility-based procedures for effective admitting and discharging the clients. Assessments of client at the time of admission to review whether care needed for a client’s condition can be provided at the facility and assessments at the time of discharge to ensure that a client with any imminent complications is not discharged into communities, are important system characteristics to improve maternal and newborn health outcomes. However, supportive services such as counselling for Family Planning and discharge counselling on danger signs did not improve significantly and this, we believe, was due to the scarcity of adequate human resources to support optimal discharge processes for mother and newborn.

An important innovation of the SCC implementation strategy in this study involved slight modification in the SCC to include documentation of maternal, foetal and newborn vital signs at key pause points followed by a provider signature. We believe that this modification of the SCC helped to promote use of the SCC at the point of care as a part of the client record and also increased provider accountability since the SCC effectively became a part of the client records. Many studies have emphasized the importance of accountability mechanisms in ensuring delivery of quality services to clients [29–31]. In the absence of robust systems to measure quality of services rendered to clients, accountability of providers to provide quality services is low in public health facilities in India. We believe that in this context, the SCC, by virtue of the mandatory recordings of the client vitals with provider signature on the SCC, has been effective in increasing the accountability of the providers which in turn has resulted in increased provider adherence with essential practices.

During the study period, a significant improvement was seen in certain practices in comparison facilities also, such as, the use of oxytocin for active management of third stage of labour, cord cutting with sterile blade and appropriate newborn thermal management. Since these practices are dependent on availability of relevant supplies, It is likely that the project component to ensure availability of essential supplies in both the intervention and comparison facilities contributed in part to this observed increased adherence with these best practices Additionally, as a part of a nursing education strengthening national initiative, in-service provider trainings in some of the comparison facilities were conducted by the government, which may explain the improvement in adherence to these practices. It is worth mentioning that among all the practices listed in the SCC, adherence with routine care practices (relevant for every mother and newborn) increased the most (such as administration of immediate post-partum oxytocin for active management of third stage of labour, initiation of immediate breast feeding). Adherence with the more complex SCC practices which require greater provider knowledge, experience and skills (e.g., resuscitation of a newborn and correct completion of the partograph) improved at a lesser rate than that for simpler routine best practices. Of note, adherence with practices rooted in social-cultural and behavioural context like hand-washing and skin-skin to contact did not improve as a result of using the checklist.

The SCC was developed by WHO to improve adherence to life saving practices in the intra- and immediate postpartum period. The SCC was piloted in one facility of Karnataka state of India in 2010, where in controlled settings, it increased adherence to some practices [22]. A similar study was conducted in one tertiary care centre of Sri Lanka where adherence to best practices as well as adherence to SCC was studied [32]. However, since both these studies were limited to just one facility and were done in controlled settings, there was a need for evidence on the effectiveness of the SCC at a scale and in resource constrained settings of developing countries like India.

The SCC, like any other checklist, is supposed to act as a reminder tool for the user, to help in minimize human errors and promote reliable human actions during complex procedures such as surgery and childbirth. However, the learning of the program in which the study was nested throws a whole new light on the way the SCC works in the developing country contexts such as India. As acknowledged globally, provider adherence to clinical practices is a function of multiple influencing factors, including provider competency and motivation and availability of essential commodities, equipment and human resources among other factors [1–4].

Two additional important determinants of adherence with best practices in the Indian context include provider accountability and nurse empowerment to participate in clinical decision making and initial management of complications. We believe that in the Indian context, the modifications in the SCC as part of the implementation strategy helped to ensure that the SCC functioned not only as a memory tool for providers but also as a framework for improving accountability (5,6) of providers due to a blended use of the SCC as checklist and a partial patient record. The adapted SCC, by virtue of being recommended by the government for use, being the part of case records,, and having added prompts for managing complications such as birth asphyxia, severe pre-eclampsia and eclampsia and post-partum haemorrhage, empowered the nurses to do initial management of maternal and newborn complications, which earlier they used to refer to doctors. Since a vast majority of vaginal deliveries are conducted by the nurses in the public health facilities, this empowerment of nurses has resulted in improved prevention and initial management of maternal and newborn complications. This mechanism of effect of the SCC has a major implication for similar resource constrained settings globally where deliveries are conducted mainly by nurses and availability of doctors is mostly sub-optimal.

Since additional programmatic components were limited to Jhpiego staff facilitating the activities, and mostly system’s resources were used for orientations and availability of any needed resources, the results of this study can be generalized to other low and middle Income countries with similar settings although further adaptation of the implementation strategy may need to be done to suit these settings. The fact that the SCC implementation strategy in this study was firmly grounded in the local public health system makes the possibility of scale up more feasible.

Key features of the implementation package that are likely generalizable to other settings include implementation within the local health system context with local system stakeholders, initial modification of the SCC based on local context, strategies to promote integration of the SCC into routine processes of maternity care (e.g., documentation of vital signs directly onto the SCC and use of the SCC as a partial individual patient record), strategies to promote provider accountability (e.g., provider signature after documentation of vital signs on SCC), empowerment of lower provider cadres to implement best practices for complications, and support for key commodities. Further research is needed into implementation strategies that may increase adherence with the most complex SCC interventions (such as complications care) and with SCC practices linked to behavioural and cultural resistance (e.g., handwashing, skin to skin care for newborn, etc.)

The study did have few limitations. Potential Hawthorne effect would have occurred due to observation of practices. But was minimized by silent observation without affecting the work there might have been some effect due to observation. But this remained the same in intervention and comparison facilities. We had a different set of observers during baseline and endline as we had nursing interns as observers. But they were trained in a standardized way using the same tools and the same trainers.

## Conclusion

Use of the SCC and provider performance of best practices increased in intervention facilities reflecting improvement in quality of facility childbirth care for women and new-born in low resource settings.

## Abbreviations

CHC: Community Health Centre
CI: Confidence Interval
DH: District Hospital
DID: Difference in Difference analysis
ENMR: Early Neonatal Mortality Rate
IMR: Infant Mortality Rate
IRB: Institutional Review Board
JSY: Janani Suraksha Yojna
MDG: Millennium Development Goals
NMR: Neonatal Mortality Rate
NRHM: National Rural Health Mission
NSSK: Navjat Shishu Suraksha Karyakram
SBA: Skilled Birth Attendants
SCC: Safe childbirth checklist
SDH: Sub-district hospital
TAG: Technical Advisory Group
WHO: World Health Organisation

## References

[1] Hogan MC, Foreman KJ, Naghavi M, Ahn SY, Wang M, Makela SM, Lopez AD, Lozano R, Murray CJ (2010). Maternal mortality for 181 countries, 1980–2008: a systematic analysis of progress towards Millennium Development Goal 5. Lancet.

[2] Lozano R, Wang H, Foreman KJ, Rajaratnam JK, Naghavi M, Marcus JR, Dwyer-Lindgren L, Lofgren KT, Phillips D, Atkinson C, Lopez AD, Murray CJ (2011). Progress towards Millennium Development Goals 4 and 5 on maternal and child mortality: an updated systematic analysis. Lancet.

[3] Rajaratnam JK, Marcus JR, Flaxman AD, Wang H, Levin-Rector A, Dwyer L, Costa M, Lopez AD, Murray CJ (2010). Neonatal, postneonatal, childhood, and under-5 mortality for 187 countries, 1970–2010: a systematic analysis of progress towards Millennium Development Goal 4. Lancet.

[4] United Nations (2014). The Millennium Development Goals Report - Addendum.

[5] Campbell OM, Graham WJ (2006). Lancet Maternal Survival Series steering group: Strategies for reducing maternal mortality: getting on with what works. Lancet.

[6] Mason E, McDougall L, Lawn JE, Gupta A, Claeson M, Pillay Y, Presern C, Lukong MB, Mann G, Wijnroks M, Azad K, Taylor K, Beattie A, Bhutta ZA, Chopra M (2014). Lancet Every Newborn Study Group, Every Newborn Steering Committee: From evidence to action to deliver a healthy start for the next generation. Lancet.

[7] Levels & Trends in Child Mortality (2013). Report 2013 eEstimates Developed by the UN Inter-agency Group for Child Mortality Estimation.

[8] Registrar General of India (2012). Sample Registration System (SRS) statistical report 2011.

[9] National institute of public cooperation and child development (2013). Statistics on Children In India.

[10] Barnes-Josiah D, Myntti C, Augustin A (1998). The “three delays” as a framework for examining maternal mortality in Haiti. Soc Sci Med.

[11] Barkat A, Rahman M, Bose ML, Com M, Akhter S (1997). Modelling the first two delays of the “three-delays model” for emergency obstetric care in Bangladesh: a choice model approach. J Health Popul Dev Ctries.

[12] Waiswa P, Kallander K, Peterson S, Tomson G, Pariyo GW (2010). Using the three delays model to understand why newborn babies die in eastern Uganda. Trop Med Int Health.

[13] Registrar General of India (2013). Sample Registration System (SRS) statistical report 2013.

[14] Ng M, Misra A, Diwan V, Agnani M, Levin-Rector A, De Costa A (2014). An assessment of the impact of the JSY cash transfer program on maternal mortality reduction in Madhya Pradesh, India. Glob Health Action.

[15] Lim SS, Dandona L, Hoisington JA, James SL, Hogan MC, Gakidou E (2010). India’s Janani Suraksha Yojana, a conditional cash transfer programme to increase births in health facilities: an impact evaluation. Lancet.

[16] Ministry of Health and Family Welfare (2014). Government of India: India New Born Action Plan.

[17] Guidelines. Government of India. http://nrhm.gov.in/nrhm-components/rmnch-a/child-health-immunization/child-health/guidelines.html. Accessed 5 Feb 2016.

[18] Nagpal J, Sachdeva A, Sengupta Dhar R, Bhargava VL, Bhartia A (2015). Widespread non-adherence to evidence-based maternity care guidelines: a population-based cluster randomised household survey. BJOG.

[19] Howitt P, Darzi A, Yang GZ, Ashrafian H, Atun R, Barlow J, Blakemore A, Bull AM, Car J, Conteh L, Cooke GS, Ford N, Gregson SA, Kerr K, King D, Kulendran M, Malkin RA, Majeed A, Matlin S, Merrifield R, Penfold HA, Reid SD, Smith PC, Stevens MM, Templeton MR, Vincent C, Wilson E (2012). Technologies for global health. Lancet.

[20] Haynes AB, Weiser TG, Berry WR, Lipsitz SR, Breizat AH, Dellinger EP, Herbosa T, Joseph S, Kibatala PL, Lapitan MC, Merry AF, Moorthy K, Reznick RK, Taylor B, Gawande AA (2009). Safe Surgery Saves Lives Study Group: A surgical safety checklist to reduce morbidity and mortality in a global population. N Engl J Med.

[21] Spector JM, Lashoher A, Agrawal P, Lemer C, Dziekan G, Bahl R, Mathai M, Merialdi M, Berry W, Gawande AA (2013). Designing the WHO Safe Childbirth Checklist program to improve quality of care at childbirth. Int J Gynaecol Obstet.

[22] Spector JM, Agrawal P, Kodkany B, Lipsitz S, Lashoher A, Dziekan G, Bahl R, Merialdi M, Mathai M, Lemer C, Gawande A (2012). Improving quality of care for maternal and newborn health: prospective pilot study of the WHO safe childbirth checklist program. PLoS One.

[23] WHO. The Safe Childbirth Checklist Collaboration. http://www.who.int/patientsafety/implementation/checklists/childbirth_collaboration_overview/en. Accessed 5 Feb 2016.

[24] Bhutta ZA, Das JK, Rizvi A, Gaffey MF, Walker N, Horton S, Webb P, Lartey A, Black RE (2013). Lancet Nutrition Interventions Review Group, Maternal and Child Nutrition Study Group: Evidence-based interventions for improvement of maternal and child nutrition: what can be done and at what cost?. Lancet.

[25] Patel A, Badhoniya N, Khadse S, Senarath U, Agho KE, Dibley MJ (2010). South Asia Infant Feeding Research Netwoork: Infant and young child feeding indicators and determinants of poor feeding practices in India: secondary data analysis of National Family Health Survey 2005–06. Food Nutr Bull.

[26] National Family Health Survey (NFHS 3) India. 2005–06 http://rchiips.org/nfhs/pdf/India.pdf. Accessed 5 Feb 2016.

[27] Pati S, Chauhan AS, Panda M, Swain S, Hussain MA (2014). Neonatal care practices in a tribal community of Odisha, India: a cultural perspective. J Trop Pediatr.

[28] Joseph N, Unnikrishnan B, Naik VA, Mahantshetti NS, Mallapur MD, Kotian SM, Nelliyanil M (2013). Infant rearing practices in South India: a longitudinal study. J Family Med Prim Care.

[29] World Bank (2004). Making Services Work for Poor People. World Development Report.

[30] Blankenberg F (2007). Taking responsibility and demanding rights: Accountability in servicedelivery.

[31] Katherine K, Paul Shaw R (2000). Reproductive Health and Health Sector Reform Linking Outcomes to Action. World Bank Institute.

[32] Patabendige M, Senanayake H (2015). Implementation of the WHO safe childbirth checklist program at a tertiary care setting in Sri Lanka: a developing country experience. BMC Pregnancy Childbirth.

